# Molecular cloning of a hyaluronidase from *Bothrops pauloensis* venom gland

**DOI:** 10.1186/1678-9199-20-25

**Published:** 2014-06-10

**Authors:** Letícia Eulalio Castanheira, Renata Santos Rodrigues, Johara Boldrini-França, Fernando PP Fonseca, Flávio Henrique-Silva, Maria I Homsi-Brandeburgo, Veridiana M Rodrigues

**Affiliations:** 1 Instituto de Genética e Bioquímica, Universidade Federal de Uberlândia, Uberlândia, MG CEP 384000-902, Brasil; 2 Department of Physics and Chemistry, School of Pharmaceutical Sciences, University of São Paulo (USP), Ribeirão Preto, São Paulo State, Brazil; 3 Department of Genetics and Evolution, Federal University of São Carlos (UFSCar), São Carlos, São Paulo State, Brazil; 4 National Institute of Sciences and Technology on Nanobiopharmaceutics (INCT), Federal University of Minas Gerais (UFMG), Belo Horizonte, Minas Gerais State, Brazil

**Keywords:** Alternative splicing, Hyaluronidase-like, Snake venom

## Abstract

**Background:**

Hyaluronate is one of the major components of extracellular matrix from vertebrates whose breakdown is catalyzed by the enzyme hyaluronidase. These enzymes are widely described in snake venoms, in which they facilitate the spreading of the main toxins in the victim’s body during the envenoming. Snake venoms also present some variants (hyaluronidases-like substances) that are probably originated by alternative splicing, even though their relevance in envenomation is still under investigation. Hyaluronidases-like proteins have not yet been purified from any snake venom, but the cDNA that encodes these toxins was already identified in snake venom glands by transcriptomic analysis. Herein, we report the cloning and *in silico* analysis of the first hyaluronidase-like proteins from a Brazilian snake venom.

**Methods:**

The cDNA sequence of hyaluronidase was cloned from the transcriptome of *Bothrops pauloensis* venom glands. This sequence was submitted to multiple alignment with other related sequences by ClustalW. A phylogenetic analysis was performed using MEGA 4 software by the neighbor joining (NJ) method.

**Results:**

The cDNA from *Bothrops pauloensis* venom gland that corresponds to hyaluronidase comprises 1175 bp and codifies a protein containing 194 amino acid residues. The sequence, denominated BpHyase, was identified as hyaluronidase-like since it shows high sequence identities (above 83%) with other described snake venom hyaluronidase-like sequences. Hyaluronidases-like proteins are thought to be products of alternative splicing implicated in deletions of central amino acids, including the catalytic residues. Structure-based sequence alignment of BpHyase to human hyaluronidase hHyal-1 demonstrates a loss of some key secondary structures. The phylogenetic analysis indicates an independent evolution of BpHyal when compared to other hyaluronidases. However, these toxins might share a common ancestor, thus suggesting a broad hyaluronidase-like distribution among venomous snakes.

**Conclusions:**

This work is the first report of a cDNA sequence of hyaluronidase from Brazilian snake venoms. Moreover, the *in silico* analysis of its deduced amino acid sequence opens new perspectives about the biological function of hyaluronidases-like proteins and may direct further studies comprising their isolation and/or recombinant production, as well as their structural and functional characterization.

## Background

“Hyaluronidase” is a term introduced by Meyer [1] to denote any enzyme that degrades hyaluronate. These enzymes are present in human testis, spleen, skin, eye, liver, kidney, uterus, placenta, tear, blood and sperm [2]. Hyaluronidases from many animal classes usually share common structural features, conserving all the critically important sites for enzyme activity [3]. Hyaluronidases were identified at first in bovine testis and in bacteria and were subsequently described as “spreading factors” [4,5].

Hyaluronidases are often found in a diversity of venoms, such as those from snakes, lizards and arthropods (scorpions, spiders, wasps and bees), in which they act as an immunogen. In snake venoms, this enzyme potentiates the toxicity and contributes to local damage at the bite site by affecting the extracellular matrix integrity due to hyaluronate degradation [6-8].

There are also some hyaluronidase variants, known as hyaluronidase-like proteins, which are products of alternative splicing [9]. These truncated forms of hyaluronidases have been recently identified in snake and bee venoms and in human serum as well [3,10,11]. Ever since, some questions have been raised about their physiological role. A hyaluronidase-like isoform from *Vespula vulgaris* venom was hypothesized to act as a lectin by binding to hyaluronate and/or other related substances without degrading them [11].

The isolation and biological characterization of hyaluronidases from snake venoms, including *Bothrops* genus, are usually difficult to achieve due to their instability and fast degradation, and because of their relatively low concentration in these biological samples. However, the purification of hyaluronidases from *Naja naja*, *Agkistrodon contortrix contortrix*, *Cerastes cerastes* and *Crotalus durissus terrificus* venoms has already been reported [12-15]. In the present work, we describe the molecular cloning and *in silico* analysis of a cDNA sequence that encodes a hyaluronidase-like protein from the *Bothrops pauloensis* venom gland. The sequence was compared to other known hyaluronidase-like sequences in order to screen conserved structural features, which may generate perspectives regarding its potential physiological functions and contributions to the envenoming.

## Methods

### Isolation of cDNA hyaluronidase from *Bothrops pauloensis* venom gland

A venom gland from a *B. pauloensis* adult snake was dissected three days after venom extraction, when transcription is most stimulated [16]. A cDNA library from *Bothrops pauloensis* venom gland, formerly referred to as *Bothropoides pauloensis*, was previously constructed by Rodrigues *et al.*[17]. Briefly, the pair of venom glands was homogenized by liquid nitrogen and the total RNA was extracted by the Trizol method (Invitrogen, UK). The mRNA was purified from total RNA by using PolyATract® mRNA Isolation kit (Promega, USA) and the cDNA library was obtained by CloneMiner cDNA Library Construction kit (Invitrogen, UK) with 3 μg of purified mRNA. First and second cDNA strands were synthesized as described by the manufacturer protocols whereas size fractioning of cDNA was carried out in a 1 mL column previously packed with Sephacryl S-500 resin. The cDNA was precipitated with ethanol and then resuspended in 50 mL of milli-Q water and submitted to Polymerase Chain Reaction (PCR). The PCR products were purified and sequenced using DYEnamic ET Terminator Cycle Sequencing kit (GE Healthcare, UK) on a MEGA-BACE 1000 automated DNA sequencer (GE Healthcare, UK).

After the cDNA sequencing, specific primers were designed in order to certify that the full open reading frame (ORF) was obtained. The specific primers were Hyase internal forward (5′-TTGGTGAAACAGCGGCCATG-3′) and Hyase internal reverse (5′-CTTTTCATCCAGCACAATAC-3′). After amplification, the PCR products were analyzed by electrophoresis on 1% agarose gel. The bands containing the PCR products were purified from gel using the Wizard SV Gel and PCR clean up system kit (Promega, Brazil), according to the manufacturer’s specifications. The Ins T/A clone PCR Product kit (Fermentas, Lithuania) was used for rapid cloning of PCR products in pTZ57R/T plasmids. Bacteria colonies were selected on a medium containing ampicillin, IPTG and X-Gal. The recombinant colonies were analyzed by PCR and gel electrophoresis. PCR products were purified and submitted to sequencing using DYEnamic ET Terminator Cycle Sequencing Kit (GE Healthcare, UK) on a MEGA-BACE 1000 automated DNA sequencer (GE Healthcare, UK). The software Base Caller Cimarron 3.12 was used to analyze the electropherograms and generate sequences, which were then aligned in the software Bioedit version 7.0.5.3.

### *In silico* analysis of cDNA sequences

Hyaluronidase sequences were searched against the NCBI database (http://www.ncbi.nlm.nih.gov/). BLASTp 2.2.19 was used for scoring the sequence alignments and the maximum e-value obtained was 2e^-100^[18]. The predicted sequence of hyaluronidase from *Bothrops pauloensis* venom and other full-length hyaluronidases and hyaluronidase-like sequences from the database were aligned by ClustalW (available in http://www.ebi.ac.uk/Tools/clustalw2/index.html). The putative glycosylation sites were identified by the glycosylation prediction software NetNGlyc (http://www.cbs.dtu.dk/services/NetNGlyc/). Predicted signal peptide cleavage site was determined by the SignalP algorithm [19]. The theoretical isoelectric point and molecular mass were computed using the tool ProtParam [20].

### Phylogenetic tree

Sequences were aligned by ClustalW algorithm and the phylogenetic analysis was performed using the software MEGA 4 by the neighbor joining (NJ) method [21]. The evolutionary distances were computed by the JTT matrix-based method. The reliability of NJ trees was evaluated by analyzing 1000 bootstrap replicates. Human hyaluronidase (NP009296.1) was employed as an out-group.

## Results and discussion

Since snakes need to kill their prey quickly and efficiently, a systemic delivery of the main venom toxins is required in order to potentiate the lethal effects. Thus, these toxins enter into the circulatory system of the victim with the aid of toxins that degrade the extracellular matrix (ECM) (metalloproteinases, myotoxic phospholipases A_2_ and hyaluronidases) [10]. Hyaluronidases have been identified in some snake venoms, such as those from *Agkistrodon acutus, Naja naja*, *Vipera russelli siamensis, Trimeresurus flavoviridis, Trimeresurus popeorum, Trimeresurus macrops, Trimeresurus albolabris, Agkistrodon contortrix contortrix* and *Crotalus durissus terrificus*[6,15,22-24].

In this study, we present the amino acid sequence of a hyaluronidase-like (BpHyase) protein deduced from a cDNA obtained from *B. pauloensis* venom gland transcriptome [17]. Interestingly, the identification of a single truncated hyaluronidase-encoding EST was achieved in an attempt to clone true hyaluronidase, which may reflect its low representation in the venom when compared to other toxin classes. On the other hand, most snake venom-gland transcriptomes reveal the presence of transcripts corresponding to hyaluronidase [10,25].

**Figure 1:**
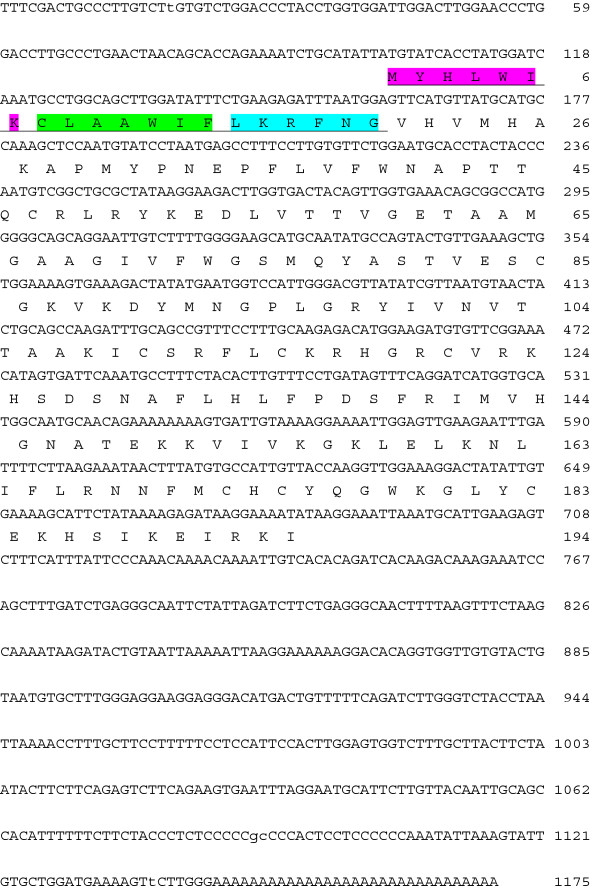
**Full-length nucleotide and deduced amino acid sequence of BpHyase.** The underlined amino acids indicate the inferred sequence of signal peptide which is characterized by an N-terminal basic region, marked in pink, a hydrophobic region (green) and a polar C-terminal (light blue) The nucleotide and amino acid sequences reported herein are available in GenBank with accession numbers GR955246 and FJ654998.1, respectively.

The cDNA sequence of hyaluronidase from *B. pauloensis* gland, denominated BpHyase, is composed of 1175 bp and codifies 194 amino acid residues for the mature protein, including eight cysteine residues (Figure 1). The full-length sequence of BpHyase comprises an ORF of 582 bp, flanked by a 5′ UTR of 100 bp and a 3′ UTR of 493 bp. The initiating methionine of BpHyase is followed by a predicted signal peptidase I (SPase I) cleavage site at FNG20-VH, which is consistent with the secreted nature of toxins. This prepeptide is believed to initiate the transport of preBpHyase into the endoplasmatic reticulum for glycosylation and is characterized by an N-terminal basic region (Met1-Lys7), a hydrophobic region (Cys8-Phe14) and a polar C-terminal (Leu15-Gly20) [26]. The presence of a prepeptide followed by a start codon indicates that BpHyase is probably translated into protein. Moreover, this prepeptide probably releases a mature protein that, after cleavage, possesses 174 amino acids with a theoretical pI of 9.60 and molecular mass of 19,892.3 Da. Therefore, this prepeptide may act as a signal sequence that directs the protein to the secretory pathway of venom gland cells [25].

**Figure 2:**
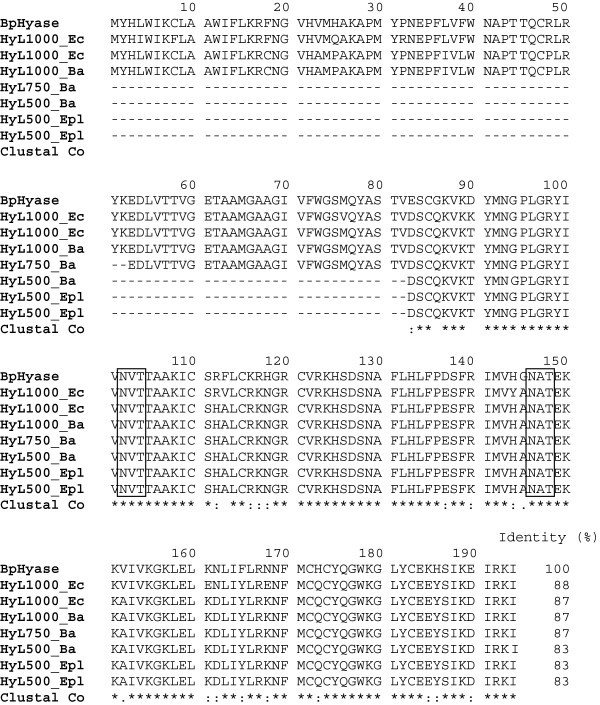
**BpHyase was aligned with truncated hyaluronidases from *Echis carinatus sochureki *(HyL1000_Ec; Genbank: ABI33950.1 and ABI33949.1), *Echis pyramidum leakeyi *(HyL500_Epl; Genbank: ABI33949.1 and ABI33942.1.), and *Bitis arietans *(HyL1000_Ba, HyL750_Ba and Hy-L-500; Genbank: ABI33947.1, ABI33946.1 and ABI33948.1) snake venoms.** The putative points of glycosylation are marked in the boxes.

The presence of N-linked glycans is supposed to be necessary for the stabilization of intramolecular folding and the consequent retention of enzymatic activity. Furthermore, changes in glycosylation are likely responsible for the diversity of biological functions exhibited by protein isoforms [27]. In relation to BpHyase, several asparagine residues identified in its sequence could potentially constitute glycosylation sites, thus influencing some physical and chemical parameters of the molecule. The glycosylation prediction algorithm (NetNGlyc) indicated the following glycosylation sites for BpHyase: N101, V102, T103 and N146, A147 and T148 (Figure 2). The glycosylation consensus triad is NXS or T, where X represents any amino acid, except proline [28,29]. However, further structural analyses are of great importance to reveal the residues truly involved in glycosylation.

Three cDNA variants of truncated hyaluronidase from *Echis pyramidum leakeyi*, *Echis carinatus sochureki* and *Bitis arietans* venom glands were already identified: Hy-L-1000 that encodes the consensus amino- and carboxy-termini with a central deletion of 256 residues, Hy-L-750 that lacks the consensus amino-terminus and Hy-L-500 that lacks the amino-terminus and encodes a shorter carboxy-terminal segment [10]. Hy-L-1000 is probably translated into a protein without enzymatic activity, while Hy-L-750 and Hy-L-500 represent non-translated transcripts due the absence of an essential translation initiating motif. The inferred protein-coding sequence of BpHyase was classified into the Glycol-Hydro-56 superfamily by protein BLAST analysis, and the highest identity (88%) was presented by truncated hyaluronidase from *Echis carinatus sochureki* venom (GenBank: ABI33950) (Figure 2). In order to confirm its identity, BpHyase was aligned by ClustalW algorithm against other reported hyaluronidase-like sequences from snake venoms, in which the highest sequence identities (above 86%) were observed for Hy-L-1000 truncated hyaluronidases, revealing that BpHyase presents the same residue deletion pattern as these molecules.

**Figure 3:**
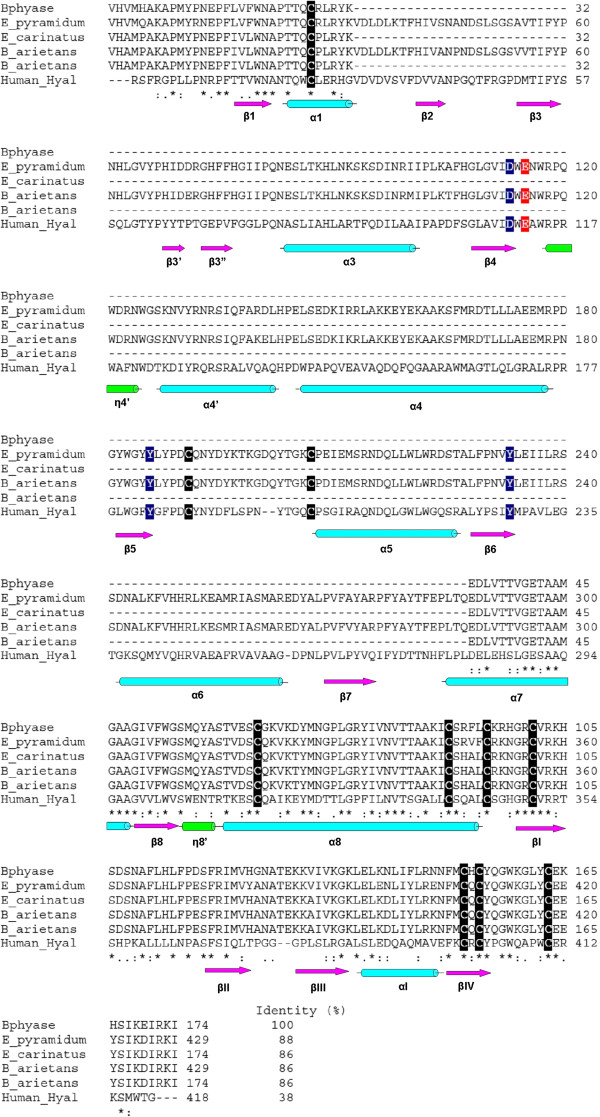
**Structure-based sequence alignment of mature truncated and complete hyaluronidases.** Full-length hyaluronidases from *Echis pyramidum leakeyi* (E_pyramidum; Genbank: ABI33941.1), Hy-L-1000 truncated hyaluronidase from *Echis carinatus sochureki* (E_carinatus; Genbank: ABI33950.1), full-length hyaluronidase from *Bitis arietans* (B_arietans; Genbank: ABI33945.1), Hy-L-1000 truncated hyaluronidase from *Bitis arietans* (B_arietans; Genbank: ABI33947.1) and human hyaluronidase (Human_hyal; Genbank: 2PE4). Key catalytic residue is shown in red and positional residues appear in blue. Cysteine residues are marked in black. Secondary structures were based on the human hyaluronidase crystal structure [38]. Secondary structure elements for human hyaluronidase are shown below the sequences: pink arrows represent β-strands, blue cylinders α-helices, and red cylinders 3_10_ helices.

It would be tempting to speculate that partial hyaluronidases or hyaluronidases-like proteins represent vestigial enzymes with no activity, since some authors affirmed they lack catalytic residues because of deletions of central residues during their evolutionary history [10,30,31]. The predicted BpHyase amino acid sequence was aligned with other full-length and truncated hyaluronidases from snake venoms, as well as human hyaluronidase (hHyal-1), in order to investigate its deletion pattern (Figure 3). The multiple alignment revealed a substantial deletion of 255 amino acids, starting at residue 52, resulting in the loss of two cysteines, the catalytic (Glu135) and positional residues (Asp133, Tyr206, Tyr253) from full-length viper hyaluronidases. Structural data, site-directed mutagenesis and steady state enzyme kinetics allowed the determination of some important residues for human Hyal-1 catalysis [32].

An essential direct role in chemical catalysis was suggested for Glu131 and a supporting role for Asp129, which was also observed by Arming *et al.*[33]. In these cases, the acidic character of the residues is critical for enzymatic activity while Glu131 acts as a proton donor to the hydroxyl group in glycosidic cleavage. These acid residues are also conserved into hyaluronidases from *Trichoderma reesei* (Glu212 and Glu217), *Bacillus agaradherans* (Glu139 and Glu228), *Echis ocellatus* venom (Asp133 and Glu 135), as well as in those from bovine testis PH20 (Asp147 and Glu 149) and *Apis mellifera* venom (Asp111 and Glu113) [34-37,10]. Moreover, Tyr202 and Tyr247 are also essential residues for catalytic activity, since Tyr202 probably binds the substrate and Tyr247 is suggested as coordinating and stabilizing the oxidation during transition state formation [32].

Figure 3 also indicates that BpHyase encodes a protein containing the consensus amino and carboxi-termini, as well as Hy-L-1000 truncated hyaluronidase variants from *E. c. sochureki* and *Bitis arietans* venoms. hHyal-1 human hyaluronidase showed a cross-generic sequence conservation of 38% when compared to BpHyase, representing the most similar hyaluronidase that had its structure solved. The alignment of hHyal-1 to BpHyase allowed the mapping of the secondary structures lost by deletions and demonstrated the lack of regions that are probably involved in the formation of β-strands 2 to 7 and α-helices 2 to 6. The crystal structures of bee venom (bvHyal) and human (hHyal-1) hyaluronidases reveal a classical (β/α)_8_ TIM barrel fold, which is common to many hydrolases [36,38,39]. In bvHyal, the barrel is formed by only seven strands (β1- β7), in contrast to hHyal-1, which presents all the eight strands [36,38]. The alignment of BpHyase and other hyaluronidases and hyaluronidases-like prteins from snake venoms with hHyal-1 demonstrates that they lack the residues responsible for forming the β-strands 2 to 7 (Figure 3). This fact indicates that BpHyase and other snake venom hyaluronidases-like proteins have a different folding pattern than that described for hHyal-1 and bvHyal, although this does not necessarily imply a loss of biological activity during envenoming.

Alternative splicing is a molecular mechanism by which different combinations of exons can be alternatively linked in order to produce different mRNA isoforms. In the globular enzymatic proteins, such as hyaluronidase, a negative selection pressure operates against gene duplication and diversification, since their correct folding is generally more sensitive to mutations [40]. Therefore, alternative splicing is the most important source of functional diversity for globular proteins in eukaryotes [41,42]. Alternative splicing variants of hyaluronidases were previously described in hHyal-1 and hHyal-3 from human prostate cancer cell lines and *Vespula vulgaris* venom [11,43]. Moreover, researchers hypothesized that spliced variants would have their function silenced and demonstrated that some hyaluronidase splicing variants from bladder tumor tissues would form a complex with true hyaluronidases, displaying the spreading activity and, consequently, regulating the functional aspects of these true hyaluronidases by alternative mRNA splicing [44,45]. Therefore, spliced variants of hyaluronidases would provide a valuable tool for modeling a metastasis inhibitor. Nevertheless, the impact of alternative splicing needs to be further investigated in hyaluronidases from human and venoms, in order to screen a biotechnological application for these recently discovered enzymes. In the present work, we suggest that BpHyase would also operate in association with a true hyaluronidase from *Bothrops pauloensis* venom in order to potentiate its activity as a “spreading factor” during the envenoming.

In addition, it was supposed that hHyal-1 splice variants may play regulatory roles by binding to partner proteins via interaction with the carboxi-terminal HyalEGF-like domain, resulting in modulation of its enzymatic activity [38]. The EGF domain is present in many extracellular proteins and is involved in cell adhesion and cell-cell communication [46,47]. In mammalian hyaluronidases, the HyalEGF-like domain is characterized by an EGF disulfide bond signature sequence. The three disulfide bonds of the HyalEGF-like domain are possibly responsible for the maintenance of its fold even when the catalytic domain unfolds [38]. Mapping deletions on the BpHyase structure demonstrates that in BpHyal-1 the segment correspondent to the HyalEGF-like domain remains intact (Figure 3), which may confer regulatory roles on these hyaluronidase-like molecules. This result is in agreement with our hypothesis that BpHyase contributes to the spreading of the main toxins into the envenomed body of the victim. On the other hand, these findings have not previously been reported for snake venoms. In this conception, further studies concerning the isolation and/or heterologous expression of hyaluronidase-like toxins would be of great interest for testifying their biological role during envenoming.

**Figure 4:**
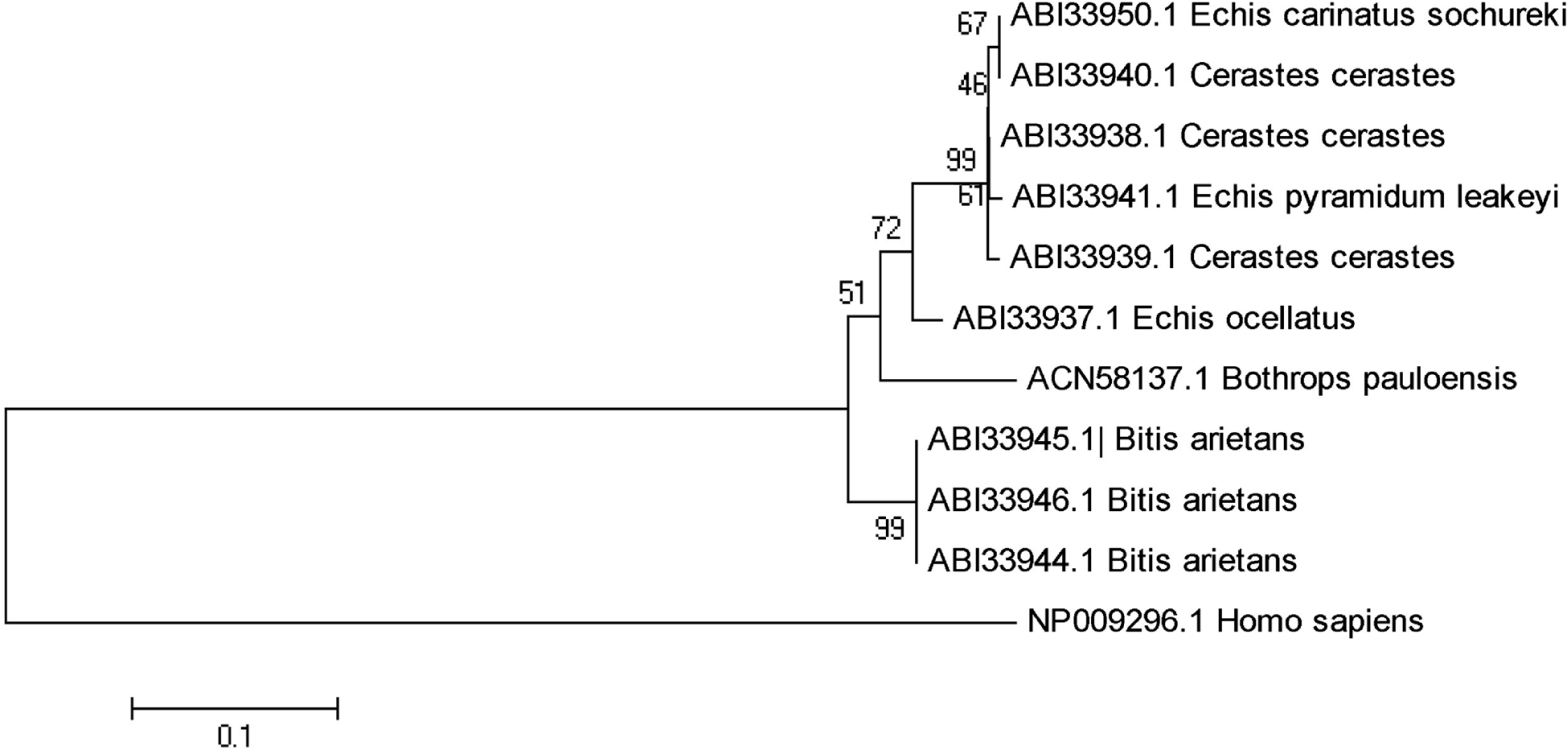
**Evolutionary relationships of snake venom hyaluronidases inferred using the neighbor-joining method.** Human hyaluronidase was employed as the out-group.

The phylogenetic analysis of hyaluronidase-like sequence and true hyaluronidases from the Viperidae family (Figure 4) showed that these molecules form a monophyletic group, indicating a recent divergence among them. The branches amongst BpHyase and other hyaluronidases may be due to point mutations and/or gene duplication, which would result in new amino acid sequences. This approach is based on the description by Futuyma [48] in which the homologous genes from different species evolve at much higher rates than others, and also, when a single gene pool changes, it can evolve into new species. BpHyase was visualized in a separate branch, which indicates an independent evolution of this toxin when compared to hyaluronidases and hyaluronidases-like proteins from *Bitis*, *Echis* and *Cerastis* genders [49]. The most interesting observation is that hyaluronidases-like proteins are, until now, described only for *Bothrops pauloensis*, *Echis carinatus* and *Bitis arietans* venoms, which inhabit different continents. All these observations suggest that hyaluronidases-like proteins may share a common ancestor, thus presenting a broad distribution among venomous snakes.

## Conclusions

Herein we have reported the first hyaluronidase-like cDNA sequence from a Brazilian snake venom. The *in silico* analysis of its deduced amino acid sequence opens new perspectives concerning its biological functions, suggesting a regulatory role that needs to be investigated. Moreover, this work may guide further studies comprising their isolation and/or recombinant production, as well as their detailed structural and functional characterization.

### Ethics committee approval

*Bothrops pauloensis* was donated by the Reptile Sector of the Federal University of Uberlândia, Minas Gerais state, Brazil. The serpentarium is registered in the Brazilian Institute of Environment and Renewable Natural Resources – IBAMA (n. 301286).

## Competing interests

The authors declare that there are no competing interests.

## Authors’ contributions

LEC, RSR and JBF contributed to the collection, analysis and interpretation of data, writing of the manuscript and its final approval. RSR participated in the collection, analysis and interpretation of data, critical reading of the manuscript and its final approval. FPPF and FHS contributed to the collection of data concerning the cDNA library and hyaluronidase cloning, critical reading of the manuscript and its final approval. MIHB and VMR were involved in critical contribution to analysis and interpretation of data, critical reading of the manuscript and its final approval. All authors read and approved the final manuscript.
